# Outcome of anal symptoms and anorectal function following two obstetric anal sphincter injuries (OASIS)—a nested case-controlled study

**DOI:** 10.1007/s00192-020-04377-3

**Published:** 2020-06-16

**Authors:** Nicola Adanna Okeahialam, Ranee Thakar, Madhu Naidu, Abdul H. Sultan

**Affiliations:** 1 Urogynaecology Clinical Research Fellow, Croydon University Hospital, Croydon, UK; 2 Croydon University Hospital, 530 London Rd, Thornton Heath, CR7 7YE UK; 3 Gynaaecare, Marathahalli, Bengaluru, India; 4 Honorary Reader at St George’s University of London, London, UK

**Keywords:** Anal incontinence, Anorectal manometry, Endoanal ultrasound, Obstetric anal sphincter injuries, Perineal trauma, Recurrent obstetric anal sphincter injury

## Abstract

**Introduction and hypothesis:**

Obstetric anal sphincter injury (OASI) is a significant risk factor for developing anal incontinence. It can therefore be hypothesised that recurrent OASI in a subsequent delivery may predispose women to further anal sphincter dysfunction.

**Methods:**

A nested case-controlled study based on data collected prospectively between 2006 and 2019. Women matched for age and ethnicity, with a history of one OASI and no sphincter damage in a subsequent delivery (control) were compared to women sustaining a second OASI. Assessment was carried out using the St Mark’s score (SMIS), anorectal manometry and endoanal ultrasound scan (findings quantified using the modified Starck score).

**Results:**

Eighty-four women were included and equally distributed between the two groups, who were followed up 12 weeks postnatally. No difference in SMIS scores was found. Maximum resting pressure (MRP, mmHg) and maximum squeeze pressure (MSP, mmHg) were significantly reduced in the study group. Median (IQR) MRP in the study group was 40.0 (31.3–54.0) versus 46.0 (39.3–61.5) in the control group (*p* = 0.030). Median (IQR) MSP was 73.0 (58.3–93.5) in the study group versus 92.5 (70.5–110.8) (*p* = 0.006) in the control group. A significant difference (*p* = 0.002) was found in the modified Starck score between the study group (median 0.0 [IQR 0.0–6.0]) and control group (median 0.0 [IQR 0.0–0.0]).

**Conclusions:**

We have demonstrated that women with recurrent OASI do not have significant anorectal symptoms compared to those with one OASI 12 weeks after delivery, but worse anal sphincter function and integrity. Therefore, on long-term follow-up, symptoms may possibly develop. This information will be useful when counselling women in a subsequent pregnancy.

## Introduction

Anal incontinence is defined as the involuntary loss of flatus or liquid and/or solid faeces [1]. Obstetric anal sphincter injury (OASI) is a significant risk factor for the development of anal incontinence, with approximately 30–40% of women developing symptoms despite primary surgical repair of the sphincter rupture following vaginal delivery [2, 3]. Anal incontinence can be both psychologically and physically debilitating and can negatively alter a woman’s quality of life [4] and subsequently affect relationships with their newborn and partners.

The estimated risk of recurrent OASI in a subsequent vaginal birth is reported in up to 10% [5, 6]. It can be hypothesised that recurrent damage potentially predisposes a woman to further anal sphincter dysfunction. Sze et al. found no significant difference in the prevalence and severity of anal incontinence among women who sustained one OASI versus two OASIs at a mean follow-up duration of 10 years [7]; however, they did not evaluate anal sphincter integrity and anorectal function.

The aetiology of anal incontinence is often multifaceted; however, normal function of the anal sphincter muscles is crucial in its maintenance. Endoanal ultrasound (EAUS) is the gold standard imaging modality for the morphological assessment of the anal sphincter [1, 8]. This is used in combination with anal manometry, clinical history and examination to assess anal sphincter function and anatomy.

The aim of this study was to evaluate anorectal symptoms, sphincter integrity and function among women who have sustained two OASIs compared to one OASI after a subsequent delivery.

## Materials and methods

This is a nested case-controlled study, comparing women who had a history of previous OASI and no sphincter damage in a subsequent delivery (control group) to women who have sustained a second OASI in a subsequent delivery (study group). These women were matched for age (age groups used: ≤ 20, 21–25, 26–30, 31–35, 36–40, < 41 years). OASIs were classified according to the Sultan classification [9]. Women were identified from a total of 1511 women in a database prospectively collected between June 2006–January 2019.

Participants underwent comprehensive postnatal assessment in a dedicated perineal clinic 12 weeks following the subsequent delivery. This included evaluation of anorectal symptoms, anal manometry and EAUS. Symptoms were assessed using the validated St Mark’s Incontinence Score (SMIS), which grades the severity of anal incontinence on a scale of 0–24 with 24 being severe incontinence [10]. EAUS was performed using the Pro-focus 2202 or Flex-focus 500 ultrasound system (BK Medical, Herlev, Denmark, type 2052; 360° rotational probe). Three-dimensional images were viewed at four levels: puborectalis, deep (proximal), superficial (mid) and subcutaneous (distal). Anal sphincter defect sizes were measured using a 3-point angle, with the centre of the anal canal forming the angle vertex. Abnormal sphincter defects detected on EAUS were quantified using the modified Starck score. An anal sphincter defect was defined as abnormal if the defect extended for > 1 h (30° angle). Images with a defect of ≤ 1 h were classified as a scar; a normal finding following primary sphincter repair [12]. The modified Starck score evaluates the extent of an abnormal anal sphincter defect diagnosed on EAUS on a scale of 0 to 16, including the length of the anal canal affected and depth and size of the defect (0 no defect – 16; > 180^0^ size defect, affecting the entire length and depth of the anal sphincter) [11].Anal manometry was performed using a validated Stryker 295-1 Intra-Compartmental Pressure Monitor [13] or the portable Anopress device (*THD* Worldwide, Correggio (RE), Italy) [14] . Maximum resting pressure (MRP) and maximum squeeze pressure (MSP) were measured. In our Perineal Clinic we follow a set protocol for the management of women in a subsequent pregnancy following OASI, which has been published previously [12]. In addition, all women and the investigations obtained are reviewed by one of two consultants experienced in pelvic floor and anal sphincter ultrasound during the perineal clinic appointment.

All data regarding history, examination, manometry and EAUS results were collected prospectively and inputted into a database as part of the normal practice for the dedicated perineal clinic at Croydon University Hospital. SMIS, anal manometry and the degree of anal sphincter defects quantified using the modified Starck score were compared between the groups.

Institutional approval was obtained but further review by a UK Research Ethics Committee was not deemed necessary.

Data were analysed using SPSS version 26.0.0.0. Descriptive statistics were calculated; the median and interquartile range (IQR) were reported. Mann-Whitney U test was used to compare data that were not normally distributed. The relationship between categorical variables was assessed using Fisher’s exact test. *p* < 0.05 was considered statistically significant.

## Results

During the study period, 84 women were included and equally distributed between the two groups. Forty-two women (study group) had sustained a second OASI in their subsequent delivery. This group was compared to 42 women (control group) who did not sustain a second OASI in the subsequent delivery. The predominant ethnic groups were Asian (43%), Caucasian (31%) and Black (26%). The overall median age was 31 (IQR 28.3–34.0) years. Women were reviewed in the perineal clinic on average 12 weeks following delivery (median 12.0 weeks [IQR 9.0–16.0]). There was no significant difference in the demographic variables or the OASI grade sustained at index delivery (during which the first OASI occurred) between the two groups (Table 1).

**Table 1:** Patient demographics

	Study group median(IQR)	Control group median(IQR)	*P* value*
Age (years)	31.0 (28.0–34.3)	32.0 (30.0–34.0)	0.563
BMI	26.0 (23.0–27.3)	26.0 (21.0–27.0)	0.469
Parity	2.0 (2.0–2.0)	2.0 (2.0–3.0)	0.609
Subsequent delivery interval (years)	3.0 (2.0–4.0)	3.0 (2.0–4.0)	0.906
Delivery to Perineal Clinic interval(weeks)	13.0 (0.8–19.5)	11.5 (9.0–15.0)	0.476
Initial OASI grade	Study group *N* (%)	Control group *N* (%)	*P* value**
3a	9 (21.4)	9 (21.4)	0.857
3b	19 (45.2)	20 (47.6)	
3c	4 (9.5)	6 (14.3)	
4th	3 (7.1)	3 (7.1)	
3rd-unclassified	7 (16.7)	4 (9.5)	

OASI, obstetric anal sphincter injury

IQR, interquartile range

*N* = number

*Mann-Whitney U test

**Fisher's exact test

Subsequent delivery details are described in Table 2. There was no significant difference in mode of delivery or mediolateral episiotomy rates at subsequent delivery. However, median infant birthweight at subsequent delivery was significantly higher in the study group (3612.5 g [3397.5–3967.5]) compared to the control (3170.5 g [2996.0–3805.0]). Table 3 describes the grade of tear sustained in the subsequent delivery.

**Table 2:** Subsequent delivery details

	Study group	Control group	*P* value
Mode of delivery (*N* [%])			
Spontaneous vaginal delivery	36 (85.7)	40 (95.2)	0.055*
Ventouse	6 (14.3)	1 (2.4)	
Forceps	0 (0)	1 (2.4)	
Mediolateral episiotomy (*N* [%])			
Yes	9 (21.4)	8 (19.0)	1.000*
No	33 (78.6)	34 (81.0)	
Birthweight g (median [IQR])	3612.5(3397.5–3967.5)	3170.5(2996.0–3805.0)	0.010**

IQR, interquartile range

*N* = number

*Fisher's exact test

**Mann-Whitney U test

**Table 3:** Subsequent delivery grade of tear

Grade of tear	Study group (*N* [%])
3a	11 (26.2)
3b	5 (11.9)
3c	10 (23.8)
4th	13 (31.0)
3rd unclassified	3 (7.1)
Grade of tear	Control group (*N* [%])
Nil	5 (11.9)
1st	5 (11.9)
2nd	32 (76.2)

*N* = number

### Symptoms

SMIS results were low and similar between the two groups at follow-up at 12 weeks. Table 4 highlights the subset scores of the SMIS. Reported faecal urgency, flatal incontinence and faecal incontinence (solid or liquid stool) were not significantly different between the two groups of women. There was also no difference in lifestyle impact.

**Table 4:** St Mark’s score breakdown

Study							
	Urge(*N* [%])	Solid(*N* [%])	Liquid(*N* [%])	Flatus(*N* [%])	Lifestyle(*N* [%])	Pads(*N* [%])	Meds(*N* [%])
Never	35.0(83.3)	40.0(95.2)	38.0(90.5)	32.0(76.2)	36(85.7)	41(97.6)	42.0(100.0)
Rarely	0(0)	1.0(2.4)	0(0)	2.0(4.8)	2(4.8)	0(0)	0(0)
Sometimes	2.0(4.8)	0(0)	1.0(2.4)	3.0(7.1)	2(4.8)	1.0(2.4)	0(0)
Usually	4.0(9.5)	1.0(2.4)	1.0(2.4)	1.0(2.4)	0(0)	0(0)	0(0)
Always	1.0(2.4)	0(0)	2.0(4.8)	4.0(9.5)	2(4.8)	0(0)	0(0)
Control							
Never	41(97.6)	42.0(100.0)	41(97.6)	37.0(88.1)	39.0(92.9)	42.0(100.0)	42.0(100.0)
Rarely	0(0)	0(0)	0(0)	2.0(4.8)	0(0.0)	0(0)	0(0)
Sometimes	1(2.4)	0(0)	1(2.4)	0(0.0)	0(0.0)	0(0)	0(0)
Usually	0(0)	0(0)	0(0)	2.0(4.8)	2.0(4.8)	0(0)	0(0)
Always	0(0)	0(0)	0(0)	1.0(2.4)	1.0(2.4)	0(0)	0(0)
P value*	*0.494*	*0.177*	*0.135*	*0.432*	*1.000*	*1.000*	*0.051*

*N* = number

*Fisher's exact test

### Anorectal function and anatomy

Compared to the control group, women with a history of a second OASI in the subsequent delivery had a significantly lower MRP and MSP. The median (IQR) MRP in the study group was 40.0 mmHg (31.3–54.0) versus 46.0 mmHg (39.3–61.5) in the control group (*p* = 0.030). Moreover, the median (IQR) MSP was 73.0 mmHg (58.3–93.5) in the study group versus 92.5 mmHg (70.5–110.8) in the control group (*p* = 0.006). There was also a significant difference (*p* = 0.002) in Starck scores between the study group(median 0.0 [IQR 0.0–6.0]) and the control group (median 0.0 [IQR 0.0–0.0]) (Table 5).

**Table 5:** St Mark’s Score, anal manometry and Starck score

	Study group Median(IQR)	Control group Median(IQR)	*P* value*
SMIS total *N* = 84	0.0(0.0–2.25)	0.0(0.0–0.0)	0.144
MRP(mmHg) *N* = 80	40.0(31.3–54.0)	46.0(39.3–61.5)	0.030
MSP(mmHg) *N* = 80	73.0(58.3–93.5)	92.5(70.5–110.8)	0.006
Starck score *N* = 84	0.0(0.0–6.0)	0.0(0.0–0.0)	0.002

IQR, interquartile range

*N* = number

SMIS, St Mark’s score

MRP, maximum resting pressure

MSP, maximum squeeze pressure

*Mann-Whitney U test

## Discussion

This nested case-controlled study of 42 women matched for age and ethnicity was designed to assess the effect of repeat OASI in a subsequent pregnancy on anorectal function. We found that 12 weeks after delivery, although there was no significant difference in anorectal symptoms, women who had sustained two OASIs had significantly reduced anal manometry pressures and more severe residual anal sphincter injury diagnosed on endoanal ultrasound compared to the controls. This study, to our knowledge, is the first study evaluating the effects of a second OASI on symptoms, anorectal function and sphincter integrity using a matched control group.

It is interesting to note that both groups of patients did not report significant symptoms including urgency or anal incontinence following the subsequent delivery as demonstrated by the low St Mark’s scores. This is probably due to the relatively short-term follow-up period of 12 weeks. Although the average subsequent delivery interval in both the control and study group was only 3 years, it is possible that on longer term follow-up the symptoms in both groups may become evident as demonstrated by studies after a single [15, 16] and after recurrent OASI [17]. Conversely, Bøgeskov et al. found no significant difference in the prevalence and severity of anal incontinence among women who sustained one OASI versus two OASIs at a follow-up duration of 10 years following OASI [7].

Although it is widely accepted that faecal incontinence is a complex, multifactorial condition, continence is partially dependent on normal sphincter anatomy and function [18]. The IAS contributes to approximately 85% of the total anal canal pressure at rest and so it is mainly responsible for anal continence at rest, the MRP [18, 19]. This is reinforced by the EAS, which supports the pressure within the anal canal and so protects continence during rises in intra-rectal or intra-abdominal pressure [20]. In addition, the EAS is responsible for voluntary anal squeeze, MSP [18]. Our study demonstrated that both MRP and MSP were significantly reduced in women with a history of recurrent OASI compared to a single OASI. This supports the findings of Jango et al. that women with recurrent OASI are more at risk of anal incontinence [17] and compromised anal sphincter function may manifest with symptoms over time.

The Sultan classification is used to grade OASIs and describes the extent of anal sphincter involvement [9, 21]. The extent of sphincter thickness involved is synonymous with the “depth of defect” subset score within the modified Starck scoring system [11]. In the present study, there was a significant difference in the modified Starck score found between women with a repeat OASI in a subsequent pregnancy compared to a single OASI. This is an important finding because in the literature it has been shown that OASIs of a higher degree are associated with worse anorectal symptoms in the long term [22]. However, there is conflicting evidence describing the effect of the degree of anal sphincter involvement diagnosed on EAUS on anorectal symptoms. In keeping with the present study findings, the severity of anal sphincter injury defined by EAUS has been described to correlate with incontinence symptoms [23, 24]. However, it is important to note that a recent large retrospective study showed that severity of anal incontinence was not associated with the extent and location of anal sphincter injury diagnosed on EAUS [25].

### Strengths and limitations

Strengths of this study include the assessment of anal incontinence symptoms and anal sphincter defects using validated tools, including the SMIS [10] and modified Starck score [11]. In addition, concerning the prospective collection of the data, to date it is the largest study of its design of women who have sustained two OASIs.

The study groups were controlled for ethnicity and age. Also, all women had subsequently achieved a vaginal delivery following index OASI. The aim of doing this was to minimise possible known and unknown confounders. Age was chosen because it has been shown to be an independent risk factor for anal incontinence in large cross-sectional studies [26, 27]. Maternal age at both first and subsequent delivery is also a risk factor for repeat OASI [5]. Ethnicity was chosen because the risk of OASI at index delivery and recurrent OASI is higher among Asian ethnic groups [5]. However, mode of delivery of the subsequent vaginal birth was not chosen because first delivery is considered the greatest risk to anal sphincter injury, as primiparous women are at an increased risk of OASI in comparison to multiparous [28]. In this study there was no significant difference in mode of delivery of the subsequent pregnancy. In addition, there was no difference in rate of mediolateral episiotomy between the study and control group, which removed mediolateral episiotomy as a potential confounder. This strengthens this study as mediolateral episiotomy has been shown to be protective against repeat OASI [5].

Although median infant birth weight was significantly higher in the study group (3612.5 g [3397.5–3967.5]), it has been described in the literature that a birthweight > 4 kg is associated with an up to three-fold increase in the risk of recurrent OASI [5, 29]. However, it has been reported that a birth weight of > 3.5 kg increases the risk of recurrent OASI by 1.5-fold [29]. The median birthweights of both groups may be expected, since our Perineal Clinic population median (IQR) birthweight from 2009 to 2019 was 3460 (3154–3760) g.

The literature shows that women with a higher grade tear are more likely to have an anal sphincter defect diagnosed on endoanal ultrasound, lower anal manometry pressures and anorectal symptoms [22, 30]. Another strength of this study is that the initial OASI tear grade was also removed as a potential confounder as there was no significant difference in OASI grade at index delivery between the two groups.

Limitations to this study include the short-term follow-up after a subsequent pregnancy. There is a need for long-term follow-up of these patients to establish the effect of two OASIs on anorectal symptoms and function, as one would expect worsening of symptoms at long term. In addition, anal manometry and endoanal ultrasound results from the index delivery where the first OASI occurred were not available for analysis. A difference in these findings may have contributed to the significant differences in the results of this study.

This nested case-controlled study has shown that, at 12 weeks, although women who sustained two OASIs had no difference in anorectal symptoms, there was worsening in anal manometry and extent of the anal sphincter defect. In the absence of randomised studies, this is the largest nested case-controlled study providing subjective and objective information following two OASIs that would be clinically useful in counselling women. This study provides further evidence to support the current recommendation that clinicians should counsel women with a history of OASI of the potential risk of new or worsening anal incontinence following a subsequent delivery. However, it is important to note that although anal sphincter anatomy is important in maintaining continence, anal incontinence is a multifactorial condition.
